# Early administration of hydrocortisone, vitamin C, and thiamine in adult patients with septic shock: a randomized controlled clinical trial

**DOI:** 10.1186/s13054-022-04175-x

**Published:** 2022-09-28

**Authors:** Qing-Quan Lyu, Rui-Qiang Zheng, Qi-Hong Chen, Jiang-Quan Yu, Jun Shao, Xiao-Hua Gu

**Affiliations:** 1 Department of Critical Care Medicine, Northern Jiangsu People’s Hospital; Clinical Medical College, Yangzhou University, Yangzhou, Jiangsu People’s Republic of China; 2 Department of Critical Care Medicine, Jiangdu People’s Hospital of Yangzhou, Yangzhou, Jiangsu People’s Republic of China

**Keywords:** Septic shock, Hydrocortisone, Vitamin C, Thiamine, Mortality

## Abstract

**Background:**

The combination therapy of hydrocortisone, vitamin C, and thiamine has been proposed as a potential treatment in patients with sepsis and septic shock. However, subsequent trials have reported conflicting results in relation to survival outcomes. Hence, we performed this randomized controlled trial (RCT) to evaluate the efficacy and safety of early combination therapy among adult patients with septic shock.

**Methods:**

This single-center, double-blind RCT enrolled adult patients with diagnosis of septic shock within 12 h from Northern Jiangsu People's Hospital between February 2019 and June 2021. Recruited patients were randomized 1:1 to receive intervention (hydrocortisone 200 mg daily, vitamin C 2 g every 6 h, and thiamine 200 mg every 12 h) or placebo (0.9% saline) for 5 days or until ICU discharge. The primary endpoint was 90-day mortality. The secondary endpoints included mortality at day 28, ICU discharge, and hospital discharge; shock reversal; 72-h Delta SOFA score; ICU-free days, vasopressor-free days, and ventilator support -free days up to day 28; ICU length of stay (LOS) and hospital LOS.

**Results:**

Among 426 patients randomized, a total of 408 patients with septic shock were included in the per-protocol (PP) analysis, of which 203 were assigned to the intervention group and 205 to the placebo group. In the PP population, the primary outcome of 90-day mortality was 39.9% (81/203) and 39.0% (80/205) in the intervention and the placebo groups, respectively, and was not significantly different (*P* = 0.86). There was no significant difference between two groups in 28-day mortality (36.5% vs. 36.1%, *P* = 0.94) or the ICU mortality (31.5% vs. 28.8%, *P* = 0.55) and hospital mortality (34.5% vs. 33.2%, *P* = 0.78). No other secondary outcomes showed significant differences between two groups, including shock reversal, vasopressor-free days, and ICU LOS. Intention-to-treat analysis included all the 426 patients and confirmed these results (all *P* > 0.05).

**Conclusion:**

Among adult patients with septic shock, early use of hydrocortisone, vitamin C, and thiamine combination therapy compared with placebo did not confer survival benefits.

*Trial registration* ClinicalTrials.gov: NCT03872011, registration date: March 12, 2019.

**Graphic Abstract:**

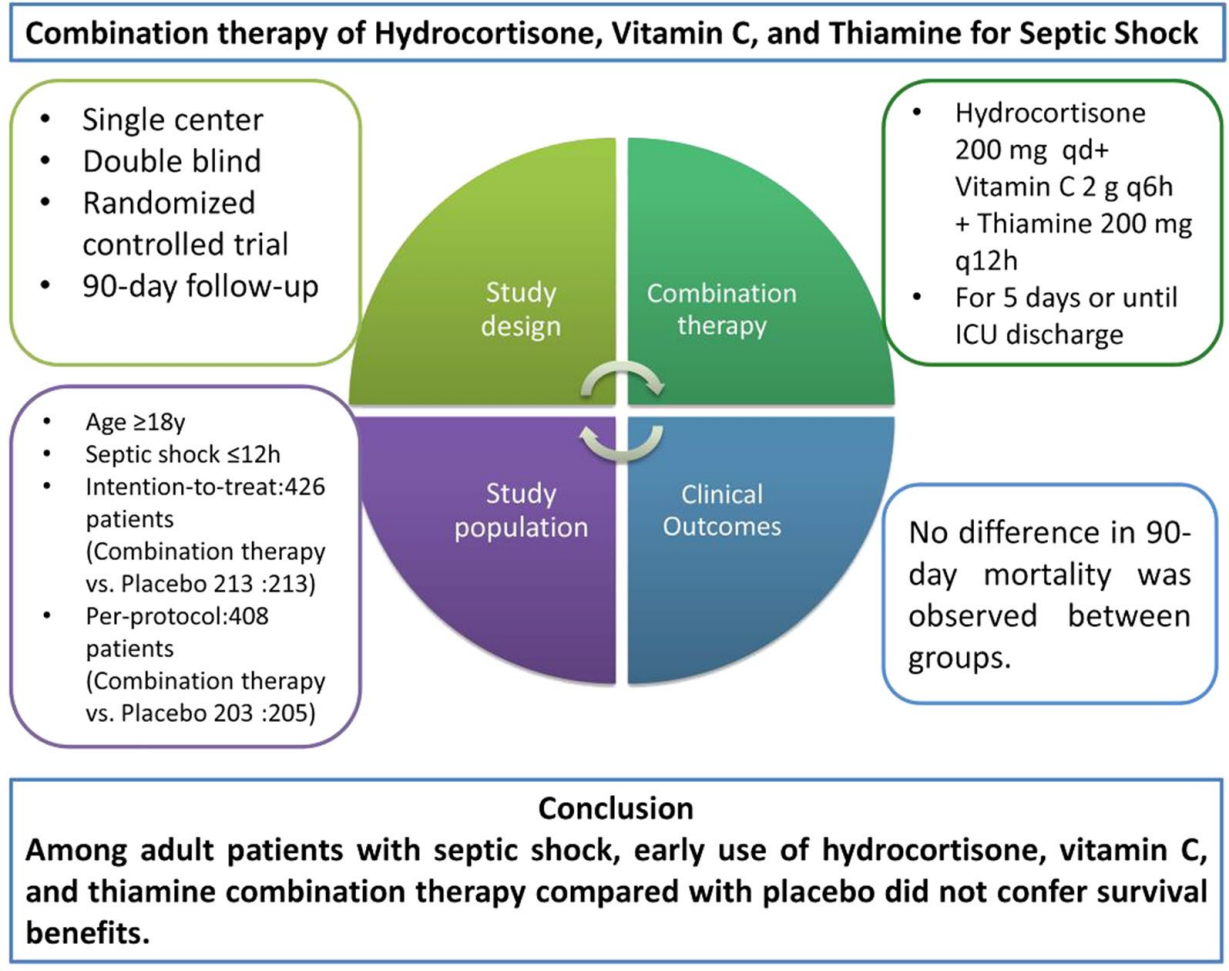

**Supplementary Information:**

The online version contains supplementary material available at 10.1186/s13054-022-04175-x.

## Introduction

Sepsis is a life-threatening condition that occurs due to a dysregulated host response to infection [1]. There were approximately 49 million cases of sepsis and 11 million sepsis-related deaths worldwide annually [2]. Septic shock, a subset of sepsis, is characterized by circulatory and cellular/metabolic abnormalities that are associated with a higher risk of mortality [3].

At present, there are no treatments directly targeting the pathogenesis of sepsis; therefore, management relies on early identification and treatment of infection through appropriate antibiotic therapy and/or source control, as well as the reversal of hemodynamic instability through fluid resuscitation and vasopressors, if necessary [3]. Therefore, safe, effective, affordable adjuvant interventions that focus on mitigating dysregulated host responses in addition to standard therapy are urgently required.

A previous retrospective before–after study of 94 patients showed that early use of combination therapy with intravenous vitamin C, hydrocortisone, and thiamine might prove to be effective in preventing progressive organ dysfunction including acute kidney injury and reducing the mortality of patients with severe sepsis and septic shock [4]. The promising results of this study have aroused great interest of the therapeutic effects of the combination therapy with vitamin C, thiamine, and hydrocortisone among sepsis and septic shock patients. However, recently published prospective, randomized controlled trials (RCTs) conducted since then reported conflicting results in relation to survival outcomes [5–8], and limited specific data are available in septic shock patients. The VITAMINS and ACTS trials, which included patients diagnosed of septic shock within 24 h, both demonstrated no significant mortality difference between the combination therapy and control groups [5, 9]. It was assumed that beneficial effect could be achieved if the combination therapy initiated early [6]. Therefore, we performed this randomized controlled clinical trial to evaluate the efficacy of early administration of hydrocortisone, vitamin C, and thiamine combination therapy for patients with diagnosis of septic shock within 12 h.

## Methods

### Study design and participants

This study was a single-center, double-blind RCT conducted in a 45-bed intensive care unit (ICU) of Northern Jiangsu People's Hospital in Yangzhou, China. The study was approved by the Human Research Ethics Committee of Northern Jiangsu People's Hospital (2019KY-145) and was registered at clinicaltrial.gov (NCT03872011). Written informed consent was obtained from patients or patients’ legally authorized representatives. Patients were enrolled from February 2019 through June 2021, with last patient follow-up in September 2021.

The inclusion criteria were as follows: (1) age 18 years old or older; (2) diagnosis of septic shock within 12 h. The exclusion criterion was the presence of any of the following: (1) systemic corticosteroid therapy within the last 3 months before septic shock; (2) high-dose steroid therapy; (3) immunosuppression; (4) pregnant; (5) known glucose-6 phosphate dehydrogenase (G-6PD) deficiency; (6) known hemochromatosis; (7) known allergy to vitamin C, hydrocortisone, or thiamine; (8) anticipated death from a preexisting disease within 90 days after randomization (as determined by the enrolling physician); and (9) refusal of the attending staff or patient family.

### Study randomization and intervention

Patients were randomized 1:1 to receive intervention or placebo. The randomization was stratified according to a table of computer-generated random numbers. Throughout the study, patients, investigators, clinical staff, and research staff remained blinded to the allocated therapy, with the exception of designated nurses who were responsible for the preparation of both study drug and placebo. The designated nurses were not involved with clinical care or outcome evaluation. Blinding regarding the trial regimen was ensured by the supply of study drug and placebo in identical, masked bags.

When the patients were diagnosed as septic shock, they were primarily treated with aggressive fluid challenge, adequate antibiotics, and vasoactive agents, according to Surviving Sepsis Campaign guidelines [3]. For the use of vasoactive drugs, norepinephrine was the first choice. At least 30 mL/kg of IV crystalloid fluid was given within the first 3 h. If target mean arterial pressure (MAP) of 65 mmHg could not be achieved, norepinephrine would be initiated within first hour of hypotension during or after 1-h fluid resuscitation. Patients in the intervention group received hydrocortisone (200 mg daily), vitamin C (2 g every 6 h), and thiamine (200 mg every 12 h) for 5 days or until ICU discharge, whichever occurred first. Vitamin C and thiamine were diluted in 100 ml 0.9% sodium chloride, respectively, and intravenously administered to patients over 60 min. Hydrocortisone was administered as a continuous infusion over 24 h. In the placebo group, an identical volume of 0.9% saline from the placebo drug bag was administered to patients using the same protocol. Attending ICU clinicians were allowed to order open-label corticosteroid therapy in place of study hydrocortisone or placebo for patients as deemed necessary (e.g., hydrocortisone for refractory shock, methylprednisolone for acute exacerbation of chronic obstructive pulmonary disease). However, the vitamin C and thiamine or matching placebo would remain randomized and blinded. These participants would remain in the study and be followed for outcomes. Other monitoring and interventions during and after the intervention period could be used in both groups at the discretion of the attending physicians.

### Definitions

Septic shock was defined as sepsis with persisting hypotension requiring vasopressors to maintain MAP ≥ 65 mmHg and having a serum lactate level > 2 mmol/L despite adequate volume resuscitation [10]. High-dose steroid therapy was defined as ≥ 2 mg/kg prednisone equivalent per day for > 5 d. Immunosuppression encompassed the following conditions: solid malignancies with a history of chemotherapy within the last 3 months, progressive metastatic disease, hematologic malignancies, solid organ transplantation, and HIV infection [11]. Length of stay (LOS) prior to randomization was defined as the time from arrival at emergency department (ED) or general ward to randomization. Diagnosis of refractory shock was dependent on the physicians’ clinical experience. Reversal of shock was defined as the maintenance of a systolic blood pressure of at least 90 mmHg without vasopressor support for at least 24 h [12]. Time to shock reversal was defined as the time from randomization to shock reversal. 72-h Delta Sequential Organ Failure Assessment (SOFA) score was calculated by subtracting the SOFA score at 72 h from the corresponding value at enrollment (ΔSOFA score = initial SOFA score at enrollment–SOFA score after 72 h). If the patient discharged within 72 h after being enrolled in the study, the SOFA score at discharge was used for the analysis. Appropriate antibiotic therapy was considered if the initially prescribed antibiotics were active against the identified pathogens, based on in vitro susceptibility testing. Fluid overload was defined as more than a 10% increase in body weight relative to baseline [(total fluid in − total fluid out) in liters/admission body weight × 100] during the course of administration of the intervention or placebo [13]. Blood gas analysis was evaluated by skilled nurses via the blood gas analyzer (Cobas b221, Roche Diagnostics); all sample tests were performed with standard factory settings. Blood glucose disturbance was considered if the blood gas analyzer reported “interference” while the same blood sample tested showed normal blood glucose reading in the central laboratory device (Cobas 8000, Roche Diagnostics), which was not affected by vitamin C.

### Data collection

The following information was collected and analyzed for every enrolled patient: age, sex, locale before ICU admission (ED/general ward), chronic medical histories (hypertension, chronic obstructive pulmonary disease, coronary artery disease, diabetes mellitus, chronic renal disease, and malignancy), primary site of infection, laboratory results (complete blood count, coagulation profile, arterial blood gas analysis, blood biochemistry), LOS prior to randomization, time from diagnosis of septic shock to randomization, time from randomization to first study drug administration, time from randomization to first antibiotic administration, proportion of antibiotic administration before randomization, appropriateness of antimicrobials, open-label corticosteroid administration, amount of fluid administered before vasopressor, ventilator support (invasive mechanical ventilation, noninvasive ventilation and high-flow nasal cannula), and renal replacement therapy (RRT) requirements. Blood culture and cultures of specimens from the site of infection were routinely performed. Disease severity was assessed by Acute Physiology and Chronic Health Evaluation II (APACHE II) [14, 15] and SOFA scores [16].

### Outcome measures

The primary outcome was all-cause mortality at day 90 after randomization. The key secondary outcomes included all-cause mortality at day 28, ICU discharge, and hospital discharge. Additional secondary outcomes included shock reversal rate; time to shock reversal; 72-h delta SOFA score; ICU-free days, vasopressor-free days and ventilator support-free days up to day 28 (patients who died before day 28 were assigned zero free days); ICU length of stay (LOS) and hospital LOS. The 90-day mortality and 28-day mortality were assessed by review of the medical records of the participant, or by contacting the participant by phone.

### Statistical analyses

We determined that a population of 406 patients (203 patients in each group) would provide the trial with 90% power to detect an absolute difference of 15 percentage points (a conservative estimate based on about 32% benefit observed in the study by Marik et al. [4]) in 90-day all-cause mortality from an estimated baseline mortality of 40%, at an alpha level of 0.05. Assuming a dropout rate of 5%, the sample size was calculated as 426 patients.

Due to the fact that most of our data were not normally distributed, which was proved by Shapiro–Wilk test and Kolmogorov–Smirnov test, we presented the data as median with interquartile range (IQR) for numerical data and numbers with percentages for categorical data. Continuous variables were compared using the Wilcoxon rank-sum test, while categorical variables were compared using the chi-square test. Patient survival time was analyzed using Cox proportional hazards regression, with results reported as hazard ratios with 95% confidence intervals (CI) and presented using Kaplan–Meier curves with a log-rank test for comparison. Proportional hazards assumptions were confirmed by Schoenfeld residuals.

Post hoc subgroup analysis for the primary outcome was performed on four subgroups determined from baseline variables, namely abdominal cavity infection, APACHE II score, age and LOS prior to randomization, with the latter three subgroups created by splitting each variable at the median level to create high and low subgroups. Every factor in subgroup analyses was analyzed with rates of the primary endpoint by testing the treatment by factor interaction with the use of Cox models.

For patients lost to follow-up, a “last status carried forward” approach would be used. And the sensitivity analyses of the primary outcome were chosen to model “Worst–Best” and “Best–Worst” scenarios: (1) Only patients lost to follow-up in the intervention group were considered to be dead; (2) only patients lost to follow-up in the placebo group were considered to be dead. Moreover, we performed the sensitivity analyses of 72-h SOFA scores to model “worst-possible” and “best-possible” scenarios: (1) The worst-possible SOFA score (score of 24) was imputed for those participants who discharged within 72 h; (2) only those patients who survive 72 h were included (Additional file 1: Tables S1 and S2). *P* values < 0.05 were considered statistically significant. Statistical analyses were performed using SPSS version 11.0.

## Results

### Patient characteristics

**Fig. 1:**
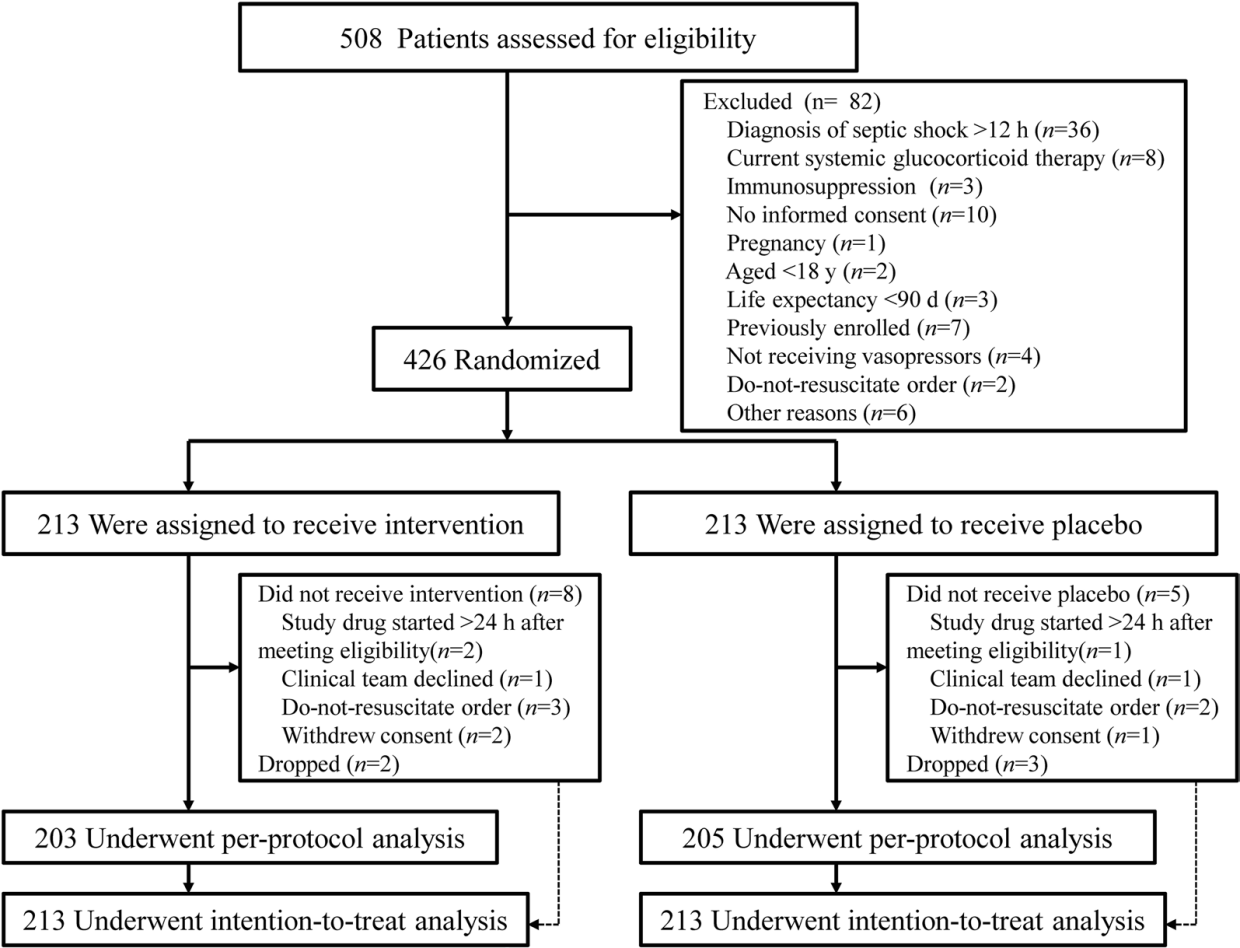
Study flowchart

A total of 508 patients were screened for this study. Of these, 82 patients were excluded; the intention-to-treat (ITT) analysis included all the 426 patients. In addition, another 10 patients in the intervention group and 8 patients in the placebo group were excluded after randomization. The specific reasons for exclusion are shown in Fig. 1. Therefore, 408 patients were included in the per-protocol (PP) analysis, of which 203 were assigned to the intervention group and 205 to the placebo group. All patients were from Chinese Han population. The baseline characteristics of the ITT and PP population are presented in Table 1. Briefly, there were no differences between the groups with respect to age, sex, APACHE II score, SOFA score, locale before ICU admission, comorbidities, and primary site of infection (*P* > 0.05).

**Table 1:** Baseline characteristics (intention-to-treat and per-protocol population)

Characteristics	Intention-to-treat analysis			Per-protocol analysis		
	Intervention (*N* = 213)	Placebo (*N* = 213)	*P* value	Intervention (*N* = 203)	Placebo (*N* = 205)	*P* value
Age (y)	69.0 (60.0–78.0)	71.0 (61.0–78.0)	0.24	69.0 (60.0–78.0)	71.0 (62.0–78.5)	0.13
Male, *n* (%)	138 (64.8)	147 (69.0)	0.35	131 (64.5)	142 (69.3)	0.31
APACHE II	20.0 (14.5–24.0)	19.0 (14.0–24.0)	0.39	20.0 (15.0–24.0)	19.0 (14.0–24.0)	0.52
SOFA	10.0 (7.0–12.0)	9.0 (7.0–11.0)	0.26	10.0 (7.0–12.0)	9.0 (7.0–11.0)	0.30
Locale before ICU admission, Emergency department, *n* (%)	87 (40.8)	73 (34.3)	0.16	82 (40.4)	67 (32.7)	0.11
At least one comorbidity, *n* (%)	146 (68.5)	153 (71.8)	0.46	138 (68.0)	149 (72.7)	0.30
Hypertension	97 (45.5)	99 (46.5)	0.85	93 (45.8)	97 (47.3)	0.76
COPD	9 (4.2)	9 (4.2)	1.00	7 (3.4)	9 (4.4)	0.62
CAD	26 (12.2)	16 (7.5)	0.10	23 (11.3)	15 (7.3)	0.16
DM	42 (19.7)	52 (24.4)	0.24	41 (20.2)	51 (24.9)	0.26
CRD	9 (4.2)	9 (4.2)	1.00	9 (4.4)	9 (4.4)	0.98
Malignancy	32 (15.0)	41 (19.2)	0.25	29 (14.3)	40 (19.5)	0.16
Primary site of infection, *n* (%)						
Lung	59 (27.7)	72 (33.8)	0.17	56 (27.6)	68 (33.2)	0.22
Abdominal cavity	120 (56.3)	103 (48.4)	0.10	113 (55.7)	101 (49.3)	0.20
Urinary tract	32 (15.0)	31 (14.6)	0.89	32 (15.8)	28 (13.7)	0.55
Skin and soft tissue	10 (4.7)	11 (5.2)	0.82	10 (4.9)	11 (5.4)	0.84
Other	21 (9.9)	20 (9.4)	0.87	20 (9.9)	20 (9.8)	0.97

*APACHE II* Acute Physiology and Chronic Health Evaluation II, *SOFA* Sequential Organ Failure Assessment, *COPD* chronic obstructive pulmonary disease, *CAD* coronary artery disease, *DM* diabetes mellitus, *CRD* chronic renal disease

Baseline clinical and laboratory measurements of the ITT and PP population are presented in Table 2. The two groups were similar in terms of baseline biological variables. Both groups had similar characteristics, such as LOS prior to randomization, time from diagnosis of septic shock to randomization, time from randomization to the first study drug administration, time from randomization to the first antibiotic administration, proportion of antibiotic administration before randomization, appropriateness of antimicrobials, open-label corticosteroid administration, amount of fluid administered before vasopressor, need for ventilator support, and need for RRT (*P* > 0.05).

**Table 2:** Baseline clinical and laboratory parameters (intention-to-treat and per-protocol population)

Parameter	Intention-to-treat analysis			Per-protocol analysis		
	Intervention (*N* = 213)	Placebo (*N* = 213)	*P* value	Intervention (*N* = 203)	Placebo (*N* = 205)	*P* value
Leucocytes (× 10^9^/L)	11.1 (4.9–17.4)	12.3 (6.5–17.0)	0.41	11.1 (4.9–17.4)	12.3 (6.6–17.0)	0.45
Platelets (× 10^9^/L)	135.0 (82.0–197.5)	143.0 (90.5–223.5)	0.10	135.0 (83.0–198.0)	143.0 (90.5–223.5)	0.14
PT (s)	15.3 (13.7–17.8)	15.5 (14.2–18.1)	0.08	15.3 (13.7–17.9)	15.5 (14.1–18.3)	0.10
PH	7.3 (7.3–7.4)	7.4 (7.3–7.4)	0.38	7.4 (7.3–7.4)	7.4 (7.3–7.4)	0.52
PaO_2_/FiO_2_ (mmHg)	240.8 (179. 5–359.5)	225.0 (159.6–344.4)	0.24	240.8 (174.0–361.0)	225.0 (159.1–344.4)	0.26
Lactate (mmol/L)	3.1 (2.3–4.4)	2.9 (2.2–4.3)	0.43	3.1 (2.3–4.4)	3.0 (2.2–4.4)	0.70
Total bilirubin (μmol/L)	17.9 (12.9–32.6)	21.9 (14.7–33.4)	0.13	17.6 (12.8–32.1)	21.9 (14.9–33.4)	0.07
Creatinine (μmol/L)	117.7 (73.0–223.5)	118.0 (76.7–209.4)	0.69	118.5 (73.0–223.5)	120.0 (76.9–211.3)	0.70
Bacteremia, *n* (%)	79 (37.1)	62 (29.1)	0.08	75 (36.9)	60 (29.3)	0.10
LOS prior to randomization (d)	0.4 (0.3–1.4)	0.5 (0.3–3.5)	0.11	0.4 (0.3–1.6)	0.6 (0.3–3.6)	0.12
Time from diagnosis to randomization (h)	2.0 (0.0–6.0)	1.5 (0.0–6.3)	0.85	2.0 (0.0–6.0)	1.8 (0.0–6.5)	0.88
Time from randomization to first study drug (h)	1.0 (0.0–3.0)	1.0 (1.0–3.0)	0.11	1.0 (0.0–3.0)	1.0 (1.0–3.0)	0.11
Time from randomization to first antibiotic (h)	2.0 (1.0–3.0)	2.0 (1.0–3.0)	0.45	2.0 (1.0–3.0)	2.0 (1.0–3.0)	0.54
Antibiotic before randomization, *n* (%)	134 (62.9)	141 (66.2)	0.48	130 (64.0)	134 (65.4)	0.84
Appropriateness of antimicrobials, *n* (%)	108 (50.7)	101 (47.4)	0.50	96 (47.3)	108 (52.7)	0.28
Open-label hydrocortisone: Additional steroids^a^	5:6	4:10	0.56^c^	5:4	4:9	0.35^c^
Amount of fluid administered before vasopressor (L)	0.8 (0.6–1.2)	0.7 (0.5–1.3)	0.56	0.8 (0.6–1.2)	0.7 (0.5–1.3)	0.71
Need for ventilator support^b^, *n* (%)	175 (82.2)	185 (86.9)	0.18	168 (82.8)	179 (87.3)	0.20
IMV: HFNC: NIV^b^	143:21:11	158:19:8	0.42	138:21:9	154:17:8	0.45
Need for RRT^b^, *n* (%)	85 (39.9)	68 (31.9)	0.09	83 (40.9)	68 (33.2)	0.11

*PT* prothrombin time, *LOS* length of stay, *IMV* invasive mechanical ventilation, *HFNC* high-flow nasal cannula, *NIV* noninvasive ventilation, *RRT* renal replacement therapy

^a^During the first 5 days after enrollment

^b^During the first day after enrollment

^c^Likelihood ratio

### Primary outcome

**Fig. 2:**
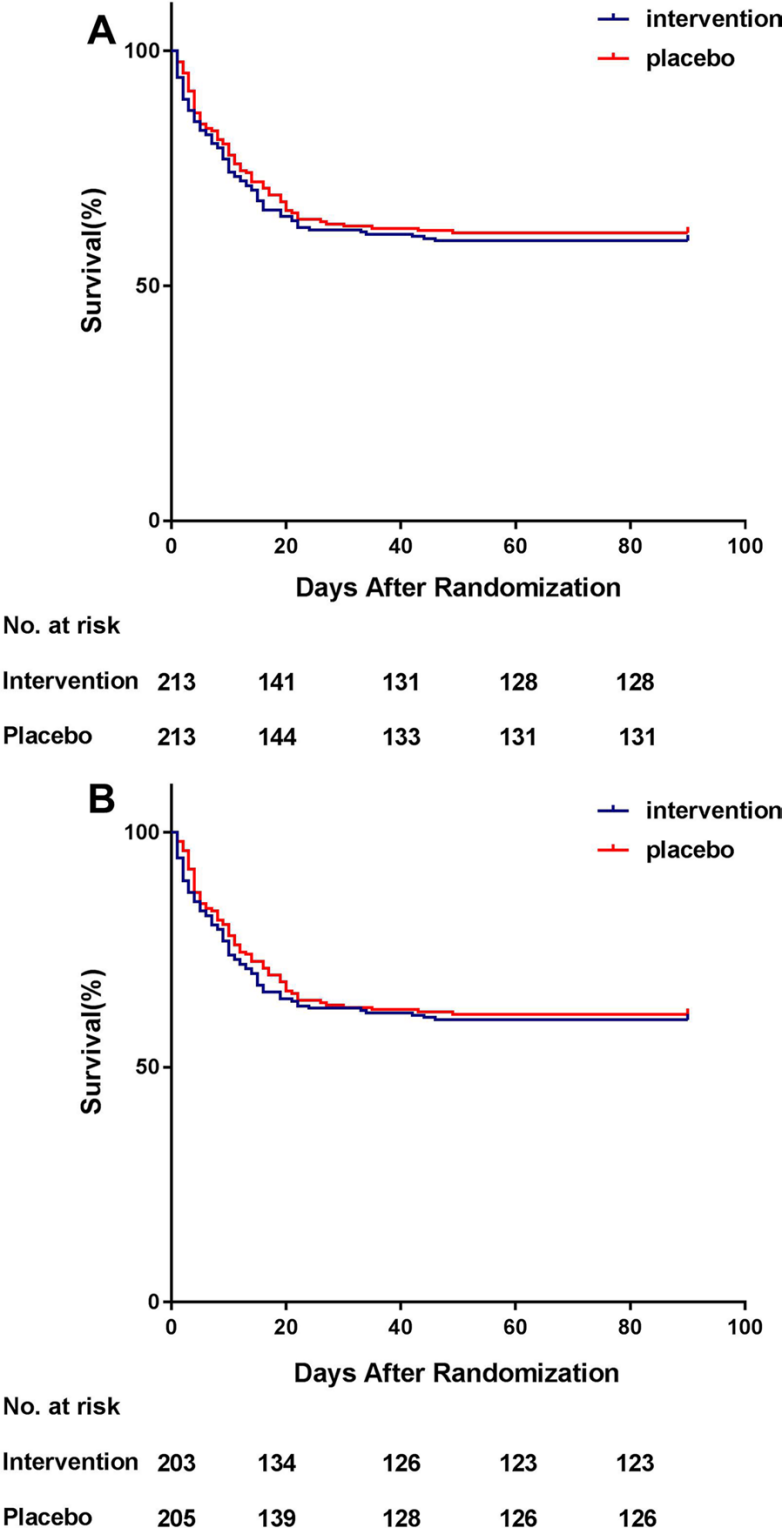
Kaplan–Meier estimates of survival rate distribution among patients in the intervention or placebo group. Panel **A** shows analysis of the intention-to-treat population. A log-rank (Mantel–Cox) test *P* = 0.62 for intergroup differences in survival rate distribution. Hazard ratio for mortality is 1.08; 95% CI 0.80–1.46. Panel **B** shows analysis of the per-protocol population. Log-rank (Mantel–Cox) test *P* = 0.67 for intergroup differences in survival rate distribution. Hazard ratio for mortality is 1.07; 95% CI 0.79–1.46

In the ITT population, the primary outcome of 90-day mortality was 40.4% (86/213) and 39.0% (83/213) in the intervention and the placebo groups, respectively, and was not significantly different (*P* = 0.77) (Table 3). In addition, the Kaplan–Meier survival curve demonstrated that the 90-day survival was not significantly different between the two groups (HR 1.08; 95% CI 0.80–1.46; *P* = 0.62) (Fig. 2).

**Table 3:** Primary and secondary outcomes (intention-to-treat and per-protocol population)

Parameter	Intention-to-treat analysis			Per-protocol analysis		
	Intervention (*N* = 213)	Placebo (*N* = 213)	*P* value	Intervention (*N* = 203)	Placebo (*N* = 205)	*P* value
90-day mortality, *n* (%)	86 (40.4)	83 (39.0)	0.77	81 (39.9)	80 (39.0)	0.86
28-day mortality, *n* (%)	79 (37.1)	77 (36.2)	0.84	74 (36.5)	74 (36.1)	0.94
ICU mortality, *n* (%)	68 (31.9)	62 (29.1)	0.53	64 (31.5)	59 (28.8)	0.55
Hospital mortality, *n* (%)	75 (35.2)	71 (33.3)	0.68	70 (34.5)	68 (33.2)	0.78
Reversal of shock, *n* (%)	172 (80.8)	174 (81.7)	0.80	164 (80.8)	168 (82.0)	0.76
Time to shock reversal (d)	3.0 (1.0–4.0)	2.0 (1.0–3.0)	0.30	3.0 (1.0–4.0)	2.0 (1.0–3.0)	0.40
72-h Delta SOFA score	2.0 (− 1.0–5.0)	2.0 (− 1.0–4.0)	0.59	1.0 (− 1.0–4.0)	2.0 (− 1.0–4.0)	0.65
ICU-free days^a^	13.0 (0.0–22.0)	14.0 (0.0–23.0)	0.67	13.0 (0.0–22.0)	14.0 (0.0–23.0)	0.23
Vasopressor-free days^a^	23.9 (0.0–26.3)	25.0 (0.0–26.5)	0.26	23.9 (0.0–26.3)	25.0 (0.0–26.5)	0.36
Ventilator support-free days^a^	17.6 (0.0–25.3)	19.3 (0.0–26.0)	0.42	17.6 (0.0–25.3)	19.6 (0.0–25.9)	0.51
LOS in ICU, days	7.0 (4.0–12.5)	6.0 (4.0–13.5)	0.85	7.0 (4.0–13.0)	6.0 (4.0–13.5)	0.93
LOS in hospital, days	16.0 (9.0–25.0)	17.0 (9.0–29.0)	0.35	16.0 (9.0–26.0)	17.0 (10.0–29.0)	0.26

*SOFA* Sequential Organ Failure Assessment*, LOS* length of stay

^a^During the first 28 days after enrollment

In the PP population, the primary outcome of 90-day mortality was 39.9% (81/203) and 39.0% (80/205) in the intervention and the placebo groups, respectively, and was not significantly different (*P* = 0.86) (Table 3). Simultaneously, the Kaplan–Meier survival curve demonstrated that the 90-day survival was not significantly different between the two groups (HR 1.07; 95% CI 0.79–1.46; *P* = 0.67) (Fig. 2).

### Key secondary outcomes

In the ITT population, there was no significant difference between the intervention and placebo groups in 28-day mortality (37.1% vs. 36.2%, respectively, *P* = 0.84) or the ICU mortality (31.9% vs. 29.1%, respectively, *P* = 0.53) and hospital mortality (35.2% vs. 33.3%, respectively, *P* = 0.68) (Table 3).

In the PP population, there was no significant difference between the intervention and placebo groups in 28-day all-cause mortality (36.5% vs. 36.1%, respectively, *P* = 0.94) or in the mortality of ICU discharge (31.5% vs. 28.8%, respectively, *P* = 0.55) and hospital discharge (34.5% vs. 33.2%, respectively, *P* = 0.78) (Table 3).

### Additional secondary outcomes

In the ITT population, the proportion of patients with reversal of shock was similar in the intervention and placebo groups (80.8% vs. 81.7%, respectively, *P* = 0.80). No significant differences were found in the time from randomization to shock reversal (3.0 vs. 2.0, respectively, *P* = 0.30); 72-h Delta SOFA score between the intervention and placebo groups (2.0 vs. 2.0, respectively, *P* = 0.59). Similarly, there was no statistically significant between-group difference in terms of 28-day cumulative ICU-free days (13.0 vs. 14.0, *P* = 0.67), vasopressor-free days (23.9 vs. 25.0, *P* = 0.26), or ventilator support–free days (17.6 vs. 19.3, *P* = 0.42) (Table 3). No other secondary outcomes showed significant differences between the intervention and placebo groups, including LOS in the ICU (7.0 vs. 6.0, *P* = 0.85) or LOS in the hospital (16.0 vs. 17.0, *P* = 0.35) (Table 3).

In the PP population, the proportion of patients with reversal of shock was similar in the intervention and placebo groups (80.8% vs. 82.0%, respectively, *P* = 0.76). There were no significant differences in the time from randomization to shock reversal (3.0 vs. 2.0, respectively, *P* = 0.40) and 72-h Delta SOFA score between the intervention and placebo groups (1.0 vs. 2.0, respectively, *P* = 0.65). Similarly, there was no statistically significant between-group difference in 28-day cumulative ICU-free days (13.0 vs. 14.0, *P* = 0.23), vasopressor-free days (23.9 vs. 25.0, *P* = 0.36), or ventilator support-free days (17.6 vs. 19.6, *P* = 0.51) (Table 3). No other secondary outcomes showed significant differences between the intervention and placebo groups, including LOS in the ICU (7.0 vs. 6.0, *P* = 0.93) or LOS in the hospital (16.0 vs. 17.0, *P* = 0.26) (Table 3).

Cox multivariate analysis demonstrated that after adjusting for potential confounding factors, age, APACHE II score, pneumonia, urinary tract infection, other sites of infection, bacteremia, time from randomization to the first antibiotic administration, lactate, need for ventilator support, and need for RRT remained independently associated with mortality (Additional file 1: Table S3).

### Adverse events

In the safety population, the most common serious adverse events were severe hypernatremia (> 160 mmol/L) (occurring in 9 patients in the intervention group and 4 patients in the placebo group, *P* = 0.16) and fluid overload (occurring in 7 and 5 patients, respectively, *P* = 0.56). In addition, blood glucose disturbance was reported in 27 patients in the intervention group (12.7% vs. 0, *P* < 0.001) (Table 4).

**Table 4:** Adverse events (safety population)

Events	Intervention (*N* = 213)	Placebo (*N* = 213)	*P* value
Severe hypernatremia, *n* (%)	9 (4.2)	4 (1.9)	0.16
Fluid overload, *n* (%)	7 (3.3)	5 (2.3)	0.56
Blood glucose disturbance, *n* (%)	27 (12.7)	0 (0.0)	< 0.001

### Subgroup analysis

In the post hoc subgroup analysis, the 90-day mortality of the all subgroups was not significantly different between the intervention and placebo groups (*P* > 0.05 for all comparisons) (Additional file 1: Fig. S1).

## Discussion

In this single-center, double-blind RCT of patients with septic shock, early initiation of hydrocortisone, vitamin C, and thiamine combination therapy compared with placebo did not significantly impact 90-day mortality and the secondary outcomes.

There is accumulative evidence indicating that vitamin C or thiamine deficiency is a common complication of septic shock patients, which is associated with immune dysfunction and poor prognosis [17]. The combination of hydrocortisone, vitamin C, and thiamine might show synergetic effect in ameliorating the systemic inflammatory response, preventing progressive organ dysfunction and reducing mortality of the septic shock patients [18].

In spite of theoretical plausibility of hydrocortisone, vitamin C, and thiamine combination therapy, our study’s results do not provide any significant survival benefit in septic shock patients, which is consistent with the results of previous RCTs [5, 6, 9, 19] and meta-analyses [20, 21]. It is worth pointing out that Marik et al. [4] and Chang et al. [22] highlighted that the early use of the combination therapy appeared to be associated with patients’ survival improvement. In our study, the diagnosis time of septic shock in included patients was within 12 h, which is earlier than previous studies [4, 22, 23]. However, early use of combination treatment in our study did not result in survival benefits.

The difference of our study from other studies should be considered when interpreting the discrepancies in efficacy of combination therapy. Firstly, the severity of organ failure is maybe a key determinant of the efficacy. The inclusion criteria of our research were restricted in patients with septic shock, a deteriorative subset of sepsis, while the research of Marik et al. [4] and Chang et al. [22] enrolled patients with sepsis and septic shock. Therefore, our included patients were more critically ill as evidenced by higher baseline SOFA scores (mean 10 points) compared with the included patients described in the research of Marik et al. (mean SOFA 8 points). Higher SOFA scores indicated an increased risk of death; it was supported by the VICTOR trial demonstrating that mortality benefit was observed only in a subset of patients with a lower SOFA score [6]. Moreover, our patients’ severity of organ failure is also corroborated by the significantly greater incidence of mechanical ventilation (72%) and the need for RRT (37%), compared with the patients included in the trial of Marik et al. (51%, 15%, respectively). In our study, the need for ventilator support and RRT was shown to be independent risk factors for mortality in patients with septic shock. And the LOVIT trial [24] also reported a high baseline severity of illness, which is maybe one of the reasons why there were no mortality benefits of vitamin C observed in the research.

Secondly, the selection of the primary endpoint should be taken into consideration. It should be pointed out that we adopted the 90-d mortality as the primary endpoint, rather than a change of the SOFA score, which was chosen as the primary endpoint in the ATESS [25], CITRIS-ALI [26], and ORANGES [27] studies. Given that the SOFA score focused on the early recovery of organ function and was assessed only if the patients remained alive during the assessment period, which could result in survivorship bias [25], the mortality endpoint was selected. However, the mortality endpoint, especially the long-term mortality, is theoretically diluted by many confounding factors [28]. However, our research did not observe any credible benefits either in non-mortality endpoints or in specific population.

Notably, our research differed from other studies in the distribution of infection sites. Abdominal infection accounted for approximately half of the cases of septic shock in this study. However, our subgroup analysis indicated that the treatment group did not show a better therapeutic effect than the control group, whether the subgroup patients had abdominal infections or not. The type of infection sites is maybe not a key determinant for the effectiveness of combination therapy. Future studies are needed to elucidate the issue.

In addition, there were no significant differences in shock reversal, or vasopressor-free days between the groups. This differs from the results of the ACTS study [9], which observed a longer shock-free days in the intervention group. In comparison with the ACTS study [9], in which hydrocortisone was given as intermittent boluses (50 mg every 6 h), hydrocortisone was given as a continuous infusion (200 mg/day) in this study. Repetitive bolus application of hydrocortisone compared with a continuous infusion likely results in higher peak serum and intracellular concentrations with greater binding to the glucocorticoid receptor and subsequently greater therapeutic effects [29]. However, hemodynamic improvement observed with the intervention in the ACTS study [9] may be related to corticosteroids alone, given that in the VITAMINS study [5], which hydrocortisone monotherapy was mandated in the control group, was not powered to detect difference in vasopressor-free days.

Intriguingly, the current study revealed a phenomenon of blood glucose measurement interference, in consistent with previous studies [30–32]. Our glucose-monitoring device adopted glucose oxidase method. Vitamin C was a strong reducing agent, thus interfering the results [32]. Our device reported “interference” reading results directly, rather than the falsely low testing results reported in some devices [30]. On the contrary, the falsely high testing results were also reported in devices adopting glucose dehydrogenase pyrroloquinoline quinone (GDH-PQQ) method [31, 33]. In this scenario, hypoglycemia may occur following insulin administration for factitious hyperglycemia [34]. Additionally, the hexokinase method was not affected by vitamin C [32]. Hence, it was adopted in our central laboratory device and recommended during the use of high-dose vitamin C.

Our study incorporates several strengths. Firstly, in terms of the timing of intervention, our study manifested an earlier application of combination therapy compared to previous studies [7, 9]. Therefore, it is possible to rule out the effect of time delay in the combination therapy application on the mortality of septic shock. Secondly, our trial investigated long-term combination therapy for septic shock. Most previous studies limited the use of combination therapy to 4 days [6, 9, 35] or even 2 days [25]. It was supposed that the lack of consistent benefits in trials mentioned above might also be due to insufficient dosage [36]. However, this trial might help to rule out the effect of duration of combination therapy on survival benefits of septic shock patients. Thirdly, to date, our trial shows a relatively larger sample size, especially compared with the trials in Chinese Han population. Fourthly, very few patients were lost to follow-up, thus minimizing attrition bias.

Some limitations of our study should be taken into consideration. First, the vitamin C and thiamine were infused over a set range of doses, and we did not measure vitamin C and thiamine levels as a guide to the dose or the duration of infusion, so it is unknown if this does/schedule was able to correct vitamin C or thiamine deficiency. Second, this was a single-center study, which may affect its external validity and generalizability.

## Conclusions

In conclusion, among patients with septic shock, hydrocortisone, vitamin C, and thiamine did not appear to reduce the 90-day mortality compared with placebo. These data do not support routine use of this combination therapy for adult patients with septic shock.

## Supplementary Information

**Additional file 1: Table S1.** Worst–Best/Best–Worst case analyses of the primary outcome. **Table S2.** Worst-possible/best-possible case analyses of the 72-h SOFA score. **Table S3.** Cox multivariate analysis of factors influencing 90-day mortality in patients. **Fig. S1.** Subgroup analysis of 90-day mortality. The forest map shows the grouped factors of the subgroup analysis, HR for 90-day mortality, 95% CI in each subgroup, and P value for interaction of treatment (intervention or placebo) and the factor. There were no significant interactions in any of the subgroups (P > 0.1 for all comparisons).

## Data Availability

The datasets used and/or analyzed in the current study are available from the corresponding author upon reasonable request.

## Abbreviations

APACHE II: Acute Physiology and Chronic Health Evaluation II
CI: Confidence interval
ED: Emergency department
GDH-PQQ: Glucose dehydrogenase pyrroloquinoline quinone
G-6PD: Glucose-6 phosphate dehydrogenase
ICU: Intensive care unit
IQR: Interquartile range
ITT: Intention-to-treat
LOS: Length of stay
MAP: Mean arterial pressure
PP: Per-protocol
RCTs: Randomized controlled trials
RRT: Renal replacement therapy
SOFA: Sequential Organ Failure Assessment
