# Supplementary material for: Early administration of hydrocortisone, vitamin C, and thiamine in adult patients with septic shock: a randomized controlled clinical trial
Source: Crit Care. 2022 Sep 28;26:295. doi: 10.1186/s13054-022-04175-x (PMC9520942; doi:10.1186/s13054-022-04175-x)
Supplement: Supplementary file 1 — Additional file 1: Table S1. Worst–Best/Best–Worst case analyses of the primary outcome. Table S2. Worst-possible/best-possible case analyses of the 72-h SOFA score. Table S3. Cox multivariate analysis of factors influencing 90-day mortality in patients. Fig. S1. Subgroup analysis of 90-day mortality. The forest map shows the grouped factors of the subgroup analysis, HR for 90-day mortality, 95% CI in each subgroup, and P value for interaction of treatment (intervention or placebo) and the factor. There were no significant interactions in any of the subgroups (P > 0.1 for all comparisons). [file 13054_2022_4175_MOESM1_ESM.docx]

**Table S1. Worst**–**Best / Best**–**Worst case analyses of the primary outcome**

| **Scenario** | **Intervention**  **(N =213)** | **Placebo**  **(N=213)** | **Log-rank**  **P value^a^** | |
| --- | --- | --- | --- | --- |
| Worst-best case analysis | | | | |
| 90-day mortality, n (%) | 88(41.3) | 83(39.0) | | 0.56 |
| Best-worst case analysis | | | | |
| 90-day mortality, n (%) | 86(40.4) | 86(40.4) | | 0.86 |

*^a^* Kaplan–Meier survival analysis

**Table S2. Worst**–**possible / best**–**possible case analyses of the 72-h SOFA score**

| **Scenario** | | **Intervention**  **(N =213)** | | **Placebo**  **(N=213)** | **P value^a^** | |
| --- | --- | --- | --- | --- | --- | --- |
| Worst-possible case analysis | | | | | | |
| 72-h Delta SOFA score | | 1.0(-3.0-4.5) | | 2.0(-2.0-4.0) | | 0.73 |
|  | **Intervention**  **(N =183)** | | **Placebo**  **(N=190)** | | **P value^a^** | |
| Best-possible case analysis | | | | | | |
| 72-h Delta SOFA score | | 3.0(0.0-5.0) | | 2.0(0.0-5.0) | | 0.84 |

*^a^* Wilcoxon rank-sum test

**Table S3. Cox multivariate analysis of factors influencing 90-day mortality in patients**

| **Variables** | **HR** | | **95%CI for HR** | | | **P value** |
| --- | --- | --- | --- | --- | --- | --- |
| Age | | 1.018 | | 1.005-1.031 | 0.006 | |
| APACHE II score | | 1.035 | | 1.012-1.059 | 0.003 | |
| Pneumonia | | 1.446 | | 1.030-2.031 | 0.033 | |
| Urinary tract infection | | 0.388 | | 0.215-0.701 | 0.002 | |
| Other sites of infection | | 1.729 | | 1.064-2.809 | 0.027 | |
| Bacteremia | | 1.405 | | 1.002-1.969 | 0.048 | |
| Time from randomization to first antibiotic | | 1.126 | | 1.035-1.225 | 0.006 | |
| Lactate | | 1.113 | | 1.066-1.163 | ＜0.001 | |
| Need for ventilator support | | 2.172 | | 1.125-4.192 | 0.021 | |
| Need for RRT | | 1.981 | | 1.406-2.790 | ＜0.001 | |

*RRT* renal replacement therapy, *HR* hazard ratio, *CI* confidence interval

**Figure S1. Subgroup analysis of 90-day mortality**

**
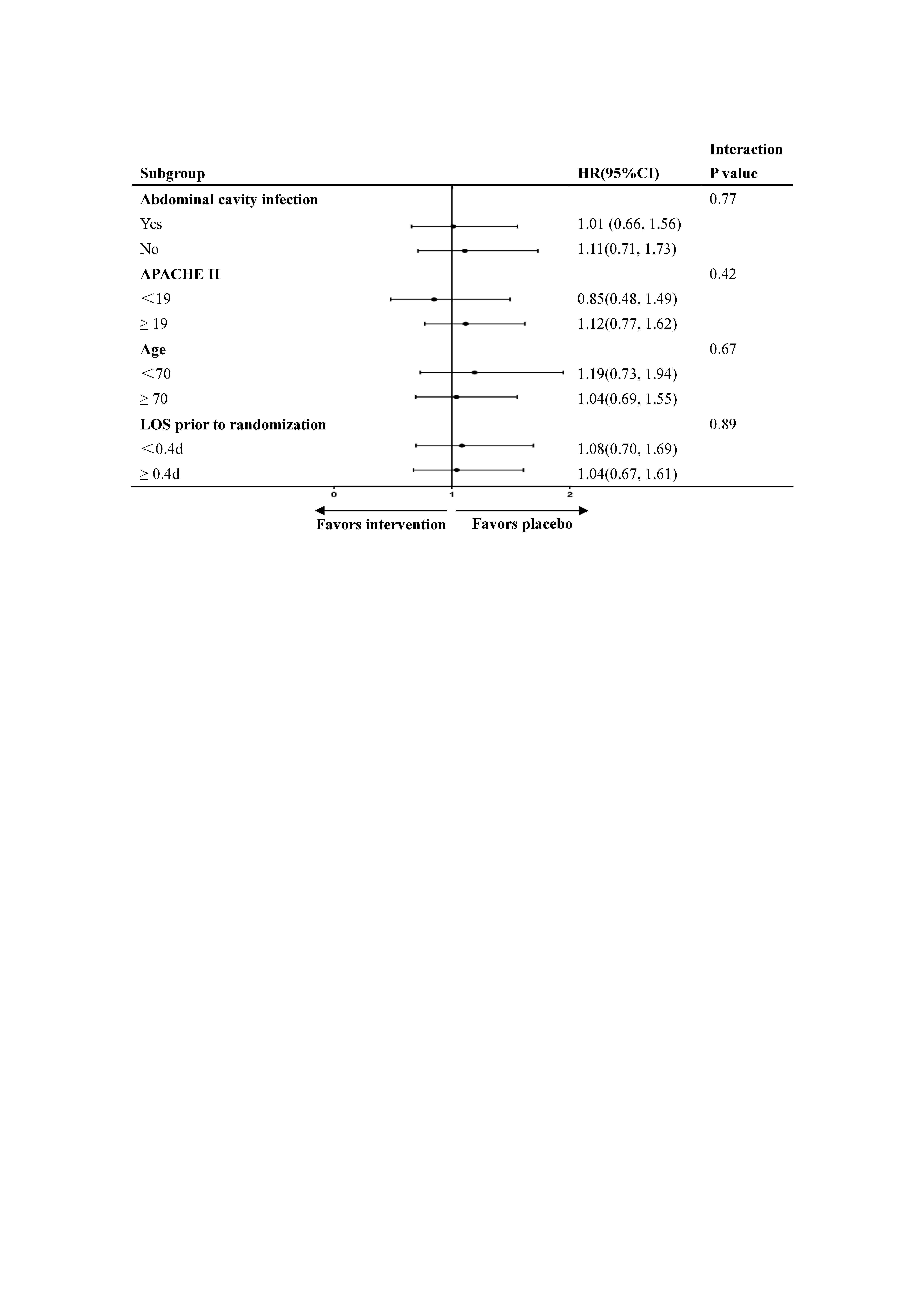
**

*HR* hazard ratio, *CI* confidence interval, *LOS* length of stay
